# Supplementary figures and images for: An update on the occurrence of Paracoccidioides species in the Midwest region, Brazil: Molecular epidemiology, clinical aspects and serological profile of patients from Mato Grosso do Sul State
Source: PLoS Negl Trop Dis. 2021 Apr 7;15(4):e0009317. doi: 10.1371/journal.pntd.0009317 (PMC8055028; doi:10.1371/journal.pntd.0009317)

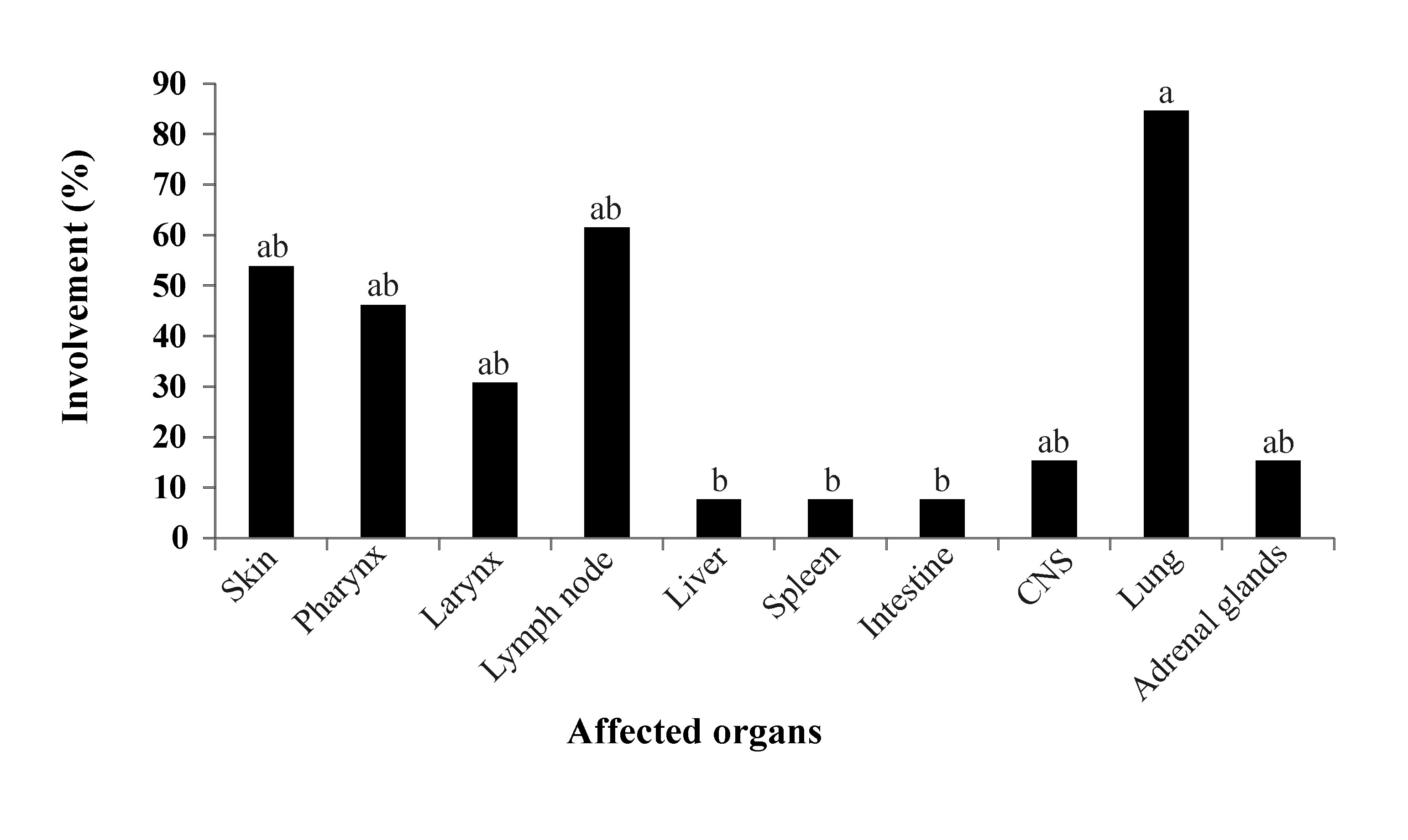

Supplement: S1 Fig — Period 2016–2019. Lower-case letters compare occurrences; frequencies followed by the same letter do not differ, while those followed by different letters are statistically different (p≤0.05). Multiple comparisons were performed using Cochran Q test. (TIF) [file pntd.0009317.s001.tif]

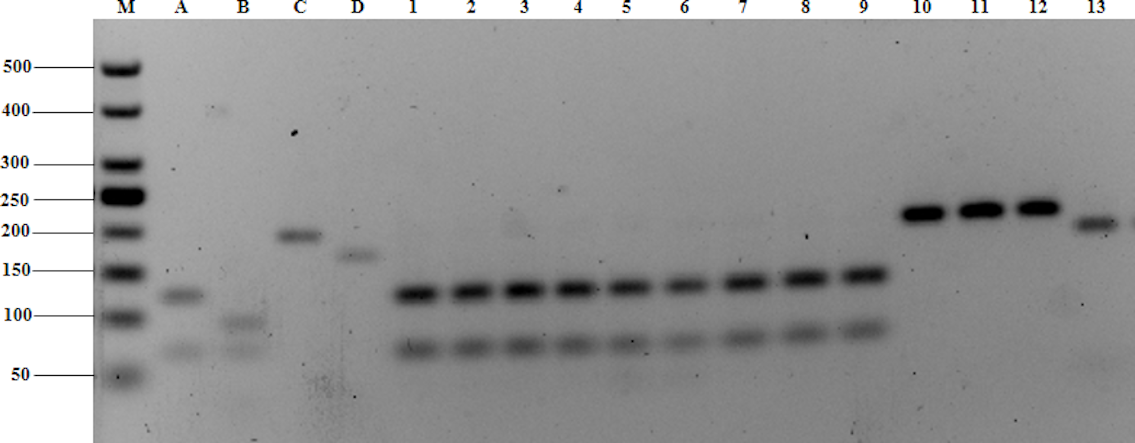

Supplement: S2 Fig — Fragment standard after digestion with BclI and MspI endonucleases (tub1 PCR-RFLP describe by Roberto et al., 2016 and modified by Hrycyk et al., 2018), showing a similarity between clinical isolates and reference strains of Paracoccidioides species. 3% agarose gel verifying similarity of fragments between the reference strains: A: Pb18—P. brasiliensis sensu stricto (S1); B: Pbdog—EPM 194—P. americana (PS2); C: EPM 54—T2—P. restrepiensis (PS3); D: Pb01—P. lutzii; and the clinical samples in this study (1 to 13—see in Table 2). M: 50 bp DNA ladder molecular weight marker (Sinapse Inc., USA). (TIF) [file pntd.0009317.s002.tif]
