# Supplementary material for: An update on the occurrence of Paracoccidioides species in the Midwest region, Brazil: Molecular epidemiology, clinical aspects and serological profile of patients from Mato Grosso do Sul State
Source: PLoS Negl Trop Dis. 2021 Apr 7;15(4):e0009317. doi: 10.1371/journal.pntd.0009317 (PMC8055028; doi:10.1371/journal.pntd.0009317)
Supplement: Attachment — Submitted filename: Response_letter.pdf [file pntd.0009317.s006.pdf]

To: Associate Editor of PLOS Neglected Tropical Diseases

Campo Grande, December 29th, 2020

Dear Prof. Dr. Claudio Guedes Salgado

We are pleased for the opportunity to submit the revised version of the article **PNTD-D-20-01595 R1** entitled “An update on the occurrence of *Paracoccidioides* species in Midwest region, Brazil: molecular epidemiology, clinical aspects and serological profile of patients from Mato Grosso do Sul State”.

We consider all editor and referees’ suggestions very constructive and helpful in improving the manuscript. We made our best effort to thoroughly incorporate all of them. Please, find below the Response Letter addressing each point raised by the reviewers.

- **Reviewer #1**.

Comments about Material and Methods:

#Methods are extremely disorganized, sorted into 3 separate sections with overlapping info that should be merged and edited for clarity.

Response: *We apologize for it. The overlapping informations were merged and the Methods was edited according suggestion, as can see in Lines 149-240.*

#Shouldn't ethics info be with patient data?

Response: *We agree with reviewer and this ethics information has been added to Lines 158-162.*

#The statistics portion is also a bit confusing- which data are being analyzed with which methods.

Response: *We agreed with the need to improve the statistics portion. Thus, this point has been addressed in Lines 232-240.*

#The information on isolates is under patients?

Response: *This topic was included in Methods in Lines 193-196.*

#It seems like the diagnostics are done with in house reagents?

Response: *Yes, until moment there are no commercial tests to PCM serological diagnosis. Then, exoantigens are frequently in house produced from standard or clinical strains and applied in routine of labs. In our case, the main strain used in Brazil is EPM01-B_339, which presents many false negative depending on the country region. Therefore, we first determined the phylogenetic species of occurrence in our state. After that, we produced exoantigens from these strains and tested by IDD method. We reinforce this information in Methods, Lines 222-230.*

#There is very little information on the serology. that was completed- is it even necessary since you retrieved the isolates?

Response: *We consider the reviewer's questioning to be extremely relevant. There are few studies showing serology data from genotyped strains and there are many cross-reactions between Paracoccidioides spp. exoantigens. Then, identify the species by molecular biology showing the serological profile is extremely relevant, and it is one of the main point of our study. Therefore, we* attached serological data as Supplementary Table 1 (Table S1) *to simplify.*

#The actual data for the strains was not presented? just the control DNA? (fig S2)

Response: *This figure was presented to demonstrate that the designed primer was able to identify Paracoccidioides fungus by using DNA controls from standard strains. However, in response to many Reviewers´ suggestions to simplify the manuscript, this PCR assay was removed and maintained results from RFLP genotyping (as seen in current Figure S2).*

Comments about Results:

#The story jumps around so much it is hard to follow. Meaningless statistical analysis on site of infection.

Response: *We appreciate the comment and apologize for it. Results were reorganized. (Lines 232-240).*

#*P. lutzii* missing from analysis in Fig 1 and 2?

Response: *In the statistical analysis description (Line 238), we include an explanation of why P. lutzii data was excluded.*

#What is the purpose of fig 3? I do not understand where these data come from

Response: *In response to many Reviewers´ suggestions to simplify the manuscript, this figure was deleted from the revised version of the manuscript.*

#Fig S1- there are multiple indications of SNPs (missing asterisk) shown that aren't SNPs? was this alignment to include *P. restrepensis*?

Response: *In response to many Reviewers´ suggestions to simplify the manuscript, this molecular assay was removed and maintained results from RFLP genotyping (as seen in current Figure S2).*

#Fig S2 would indicate that *P. restrepensis* does not amplify a 303bp fragment.

Response: *In response to many Reviewers´ suggestions to simplify the manuscript, this molecular assay was removed and maintained results from RFLP genotyping.*

Comments about Conclusions:

The authors admit that only 20% of the patient samples grew isolates, but fail to recognized that the failure to grow in culture could be species specific- e.g. perhaps *P. lutzii* does not grow as well. How does understanding species in the region change treatment or diagnostic approaches. It is sort of addressed starting at line 403, but the data presented in the paper don't clearly support the claim because the numbers are not there to produce a convincing result (n=1 for P.l.). All other case data simply support previous observations.

Response: We consider extremely relevant these comments. Indeed, *P. lutzii* could have more difficulty in growth *in vitro*, which could be associated with the only isolation in this study. In addition, we agree that the sample number is very small to make any hypothesis in this moment. However, due the isolation of fungus from culture to be an infrequent event, these clinical, serological and molecular data publication could be considered, in order to define the present picture and future directions, according reported in Discussion (Lines 349-354).

Comments about Editorial and Data Presentation Modifications: The methods section needs to be extensively revised. The authors need to clearly assess which data are new and focus on that part of the story. Much of the case data could be combined to a single table, and possibly be in supplemental.

Response: As suggested by the reviewer, we reorganized the Methods (Lines 185-240). In addition, serological and clinical data were attached as supplementary material (Figure S1 and Tables S1-S2). Only original data about genotyping of *Parcoccidioides* species in Midwest region were kept in the main text.

Comments about Summary and General Comments: The study presents information on species distribution of the causative agents of PCM in central Brazil, which represents 3 different species of *Paracoccidioides*. Only 20% of the patients produced cultures from samples. Some of these patients had negative serology, but the generalization of this observation is complicated by small sample size. The clinical significance is not made clear- are there differential outcomes for these patients? differing treatment regimes? There are some good points in the discussion, but the overall lack of clarity in the presentation makes it impossible to see the main, and important, conclusions.

Response: *We understand the reviewer's opinion and thoroughly revised the discussion (Lines 340-456) in order to improve this point. We hope that the new version is suitable.*

- **Reviewer 2**.

Comments to the authors:

#The clinical presentations of patients are too heterogeneous to allow adequate comparisons to be made.

Response: *We agree that the limitation of this study really is the small number of patients / isolates evaluated. Here, the statistical analysis applied comparing serological and clinical data of P. brasiliensis and P. restrepiensis, did not allow defining clinical differences. However, the continuous character of this procedure may be considered, in order to define the present picture and future directions. Despite that, identify species by molecular approach showing the clinical and serological profile of patients is relevant, and it is one of the main point of our study. In this sense and in response to many Reviewers´ suggestions to simplify the manuscript, the serological and clinical data were attached as supplementary material (Figure S1 and Tables S1-S2). Only data about genotyping of Parcoccidioides species in Midwest region were kept in the main text.*

#It is very interesting how the authors took the migratory history of the patients. Since the majority of patients had chronic forms of PCM it is difficult to do a georeferencing analysis.

Response: *We agree with the punctual comments of reviewer. We really can not say with accuracy the place of Paracoccidiodes spp. infection. Therefore, we consider interesting at least to describe the place of birth and current residence, and we were careful to make this clear in the Method (Lines 176-179).*

#The authors conducted a review of the literature and present a map with the updated geographical distribution of PCM species.

Response: *Yes, the Figure 2 and Table S3 presents an update on the Paracoccidioides species distribution in the Midwest region of Brazil. Because, this study records for the first time molecular data about Paracoccidioides isolates in the state of Mato Grosso do Sul, the only state of this region with no previous records.*

#A statistical inference analysis of the 13 patients was performed. The sample is small and the results may have little power of statistical inference. More research is required on the geographic spread of PCM species and the clinical and epidemiological differences.

Response: *We agree that the limitation of this study really is the small number of patients / isolates evaluated, and that more studies are required on the geographic spread of PCM species to determine clinical and epidemiological differences. However, the continuous character of this procedure could be considered, in order to define the present picture and future directions, according reported in Discussion (Lines 351-354).*

#As a request, I would like you to review figure 4, since the legend is not clear enough and it seems that a mistake was made with the symbols used: triangles (PS1), circles (PS3), squares (PL).

Response: *We appreciate the indication of the editing mistake in the symbols used in Figure 4 (named as Figure 2 in current version of manuscript). We apologize for it. Now, legend and figure are edited correctly.*

- **Reviewer 3**.

Comments about Methods:

#Methods are extensively described. Description of PCM clinical forms is unnecessary in the context of your results and the low number of cases of whom 12/13 presented the chronic clinical form.

Response: *As suggested by reviewers, we reorganized the Methods and description of PCM clinical forms was just cited (Lines 173-174).*

#Molecular methods would be only referenced because they were already full described.

Response: *As suggested by the reviewer, the molecular methods were only referenced. (Lines 207-210).*

#The description of the Fava Neto medium composition is enough known and can be removed.

Response: *We agree with the reviewer and this information were removed from the manuscript (Lines 191).*

Comments about Results:

#There are several tables and figures which could be removed such as: Table 1; table 2 ; Table 4 ; Figure 1, Figure 2, figure 3. This suggestion is due their repetitive information and low contribution in the context of 13 cases of PCM. Maybe one or two tables or figures can condense the data more relevant.

Response: *We agree with the reviewer. We reorganized the results, serological and clinical data were condensed and attached as supplementary material (Tables S1-S2 and Figure S1).*

#It would be very interesting to present the dendogram of these species.

Response: *We appreciate this suggestion. The dendrogram is presented as Figure 1 in the new version of the manuscript.*

Comments about Conclusions:

#I suggest that the discussion be more focussed on molecular aspects of these species than on clinical or serological data due to the low number of cases presented and most related data are enough known in the literature on PCM.

Response: *As suggested, we present a new version of the discussion (Lines 340-456), in which we tried to incorporate the recommendations, emphasizing molecular findings.*

#I suggest to remark the relevance of plotting the geographical distribution of Paracoccidiodes spp in the latin American context where PCM is endemic and the potential to establish their relation with clinical forms, host preference, therapeutical outcome and balance infection/disease among others.

Response: *As suggested, we present a new version of the discussion (Lines 340-456), in which we tried to incorporate the recommendations, emphasizing molecular findings.*

#Line 357: you stated that there are geographical expansion of *Paracoccidiodes* spp. what is the evidence about? Maybe and currently, molecular tools permit us to know how *Paracoccidiodes* species are distributed in areas as Mato Grosso, classically considered endemic to PCM. What do you think about?

Response: *We agree that we cannot affirm that there was geographical expansion of Paracoccidiodes spp., only describe the distribution of species in the studied area. For it, we would need to do studies comparing between clinical and environmental strains. We apologize for the mistake, and we edited the paragraph in the manuscript (Lines 362-366).*

Comments about Editorial and Data Presentation Modifications: Along the text there are several premises or words which could be avoided or changed such as:

Please avoid as possible words as high prevalence, higher incidence, few adequates in the PCM context.

Response: The words *were changed in lines 357 and 378, according suggested.*

#Line 39; which is one of the most instead the most important.

Response: *It was changed in Line 69.*

#Line 44: accuracy instead efficacy.

Response: *The paragraph was deleted from the current manuscript.*

#Line 45: Paracoccidiodes spp instead PCM.

Response: *It was changed in Line 110.*

#Line 57: a technique that help to answer questions about epidemiological. It is unclear.

Response: *We agreed that this was unclear. We apologize for it, and the phrase was edited (Line 122).*

#Lines 66-69: But low incidence of the etiologic agent. These sentences are confuses

Response: *We agree and apologize for the confuse. The phrase was edited in the resubmitted report (Lines 130-133), in order to improve clarity.*

#In general the text is very extensive with a lot of information already described by others along the time. I suggest it be more concise in order to catch the reader attention on molecular data.

Response: *As suggested, we present a new version more concise of the manuscript, which we tried to incorporate the recommendations, emphasizing molecular findings.*

Comments about Summary and General Comments: I carefully read your manuscript, which contents novel and interesting data on *Paracoccidiodes* spp in Mato Grosso state of Brazil. My main concern is related with its extensive form with information already described. I remark the relevance of your results which would be more explored on the molecular and geographical context than on serological and clinical ones. In my opinion, focus on molecular issues is pivotal in your paper.

Response: *As suggested, we present a new version of the manuscript, which we tried to incorporate the recommendations, emphasizing molecular findings.*

We hope the current version of the manuscript will meet the standard of PLOS Neglected Tropical Diseases.

Sincerely,

Simone Schneider Weber (corresponding author)
